# Management of Obstructive Sleep Apnea in Patients With Heart Failure

**DOI:** 10.3389/fmed.2022.803388

**Published:** 2022-02-18

**Authors:** Youmeng Wang, Christoph Schöbel, Thomas Penzel

**Affiliations:** ^1^Sleep Medicine Center, Charité-Universitätsmedizin, Berlin, Germany; ^2^Universitätsmedizin Essen, Ruhrlandklinik - Westdeutsches Lungenzentrum am Universitätsklinikum Essen GmbH, Essen, Germany

**Keywords:** heart failure, treatment, obstructive sleep apnea, central sleep apnea, PAP

## Abstract

Sleep apnea is traditionally classified as obstructive sleep apnea (OSA), which occurs when the upper airway collapses due to the relaxation of oropharyngeal musculature, and central sleep apnea occurs when the brainstem cannot stimulate breathing. Most sleep apnea in patients with heart failure (HF) results from coexisting OSA and central sleep apnea (CSA), or complex sleep apnea syndrome. OSA and CSA are common in HF and can be involved in its progression by exposure to the heart to intermittent hypoxia, increased preload and afterload, activating sympathetic, and decreased vascular endothelial function. A majority of treatments have been investigated in patients with CSA and HF; however, less or short-term randomized trials demonstrated whether treating OSA in patients with HF could improve morbidity and mortality. OSA could directly influence the patient's recovery. This review will focus on past and present studies on the various therapies for OSA in patients with HF and summarize CSA treatment options for reasons of reference and completeness. More specifically, the treatment covered include surgical and non-surgical treatments and reported the positive and negative consequences for these treatment options, highlighting possible implications for clinical practice and future research directions.

## Introduction

Heart failure (HF) is a common clinical syndrome of insufficient cardiac output to meet physiological requirements that carry high levels of mortality and morbidity (1). Because of repeated hospitalizations, decreasing productivity, and medical treatment fees, HF has a substantial economic impact on the community (2). The leading causes of HF are coronary artery disease, myocardial infarction, and cardiomyopathy, which can impair the structure and function of cardiac ventricles (3). Obesity, diabetes mellitus, hypertension, smoking, alcohol abuse, anemia, thyroid dysfunction, and atrial fibrillation are common risk factors for HF (4). HF is also accompanied by chronic kidney failure, malabsorption, depression, and anorexia nervosa (5). In the developed countries, about 1% of the population suffers from HF, with an increasing prevalence to around 9% in those >70 years (6). The main medication for HF include diuretics, beta-blockers, angiotensin-converting enzyme inhibitors (ACEIs), angiotensin receptor blockers (ARBs). Research showed medications had improved 5-year survival from about 45 to 70% (7, 8). In recent years, device treatments such as a defibrillator and pacemaker therapy, and left ventricular assist devices (LVADs) have improved survival (9).

Sleep disorder breathing (SDB) is more prevalent in HF patients than in any other population. A previous study showed that ~75% of patients with HF have SDB. It is associated with sleepiness, chronic bronchitis, peripheral edema, and dyspnea (10). SDB could be mainly classified into two types, obstructive sleep apnea (OSA) and central sleep apnea (CSA) (11). OSA is an increasingly prevalent sleep disorder characterized by repeated episodes of upper airway collapse and obstruction during sleep, associated with breathing pauses or oxygen desaturation (12). Abnormalities are led to untreated OSA with hypercapnia, contributing to behavioral and cognitive impairment, metabolic syndrome, and cardiovascular (CV) diseases (13). Around 33% of men and 16% of women between the ages of 35 and 75 years have OSA (14). Due to a lack of awareness of patients and their doctors, a large number of patients with moderate or severe OSA may still not be diagnosed and treated (15). Stigma related to snoring and the poor availability of centers that conduct polysomnography (PSG) studies, these factors make OSA challenging to diagnose and treat (16). OSA is one of the common complications in patients with HF, and its prevalence ranges between 60 and 78% (17). CSA is a frequent finding in HF, both in those with reduced ejection fraction and preserved ejection fraction (18–20). The presence of CSA appears to have adverse prognostic implications (21, 22). It is important to note that HF results from coexisting OSA and CSA, or complex sleep apnea syndrome (23). However, patients have little understanding of coexistence. There is no OSA screening program for HF patients, caused by the high cost of polysomnography and the limited availability of sleep centers even in the developed community (24). Although there is a strong link between HF and OSA, randomized controlled studies have failed to show that sleep disturbance treatments might enhance CV disease outcomes in individuals with diagnosed HF (25, 26).

OSA prevalence in patients with HF with systolic dysfunction ranged from around 20–45% (27). Severe OSA with an apnea-hypopnea index (AHI) >20 events/h was shown to be prevalent in around 26% of people. Male HF patients with a body mass index (BMI) >32 kg/m^2^ have a higher incidence of OSA (28). On the other hand, BMI is not a valuable indication for women. Women over 65 are six times as likely as young women to suffer from OSA (28). Due to greater clinician understanding and widely disseminated guidelines, the percentage of HF patients on evidence-based therapy has increased over the last decade (29). Despite increased usage of β-blockers and aldosterone antagonists, the prevalence of OSA in patients with HF has remained unchanged (20). This implies that treating HF alone is not enough in this demographic (population) (30). Craniofacial or oropharyngeal anatomic anomalies, male sex, obesity, and smoking are significant risk factors for OSA (13). The dilator muscles' tone that keeps the airway patency decreases during sleep. Genioglossus muscle relaxation, in particular, permits the tongue to fall posteriorly inside the throat, allowing obstruction in those who are vulnerable (31). Obesity and other anatomic variables cause a narrowing of the airway lumen to enhance the chances of obstruction (32). OSA affects non-obese people with no obvious anatomic anomalies, suggesting that non-anatomic causes are equally significant. Two examples are instability in ventilatory control and a lower sleep arousal threshold (33). Individual patients' relative contributions from these processes differ, which could have therapeutic consequences. In the future, more excellent knowledge of the mechanisms underlying OSA may allow for more tailored therapy strategies (32). The prevalence of CSA in healthy adults appears to be lower than that of OSA, but there is no strong evidence for this issue so far (34).

**Figure 1:**
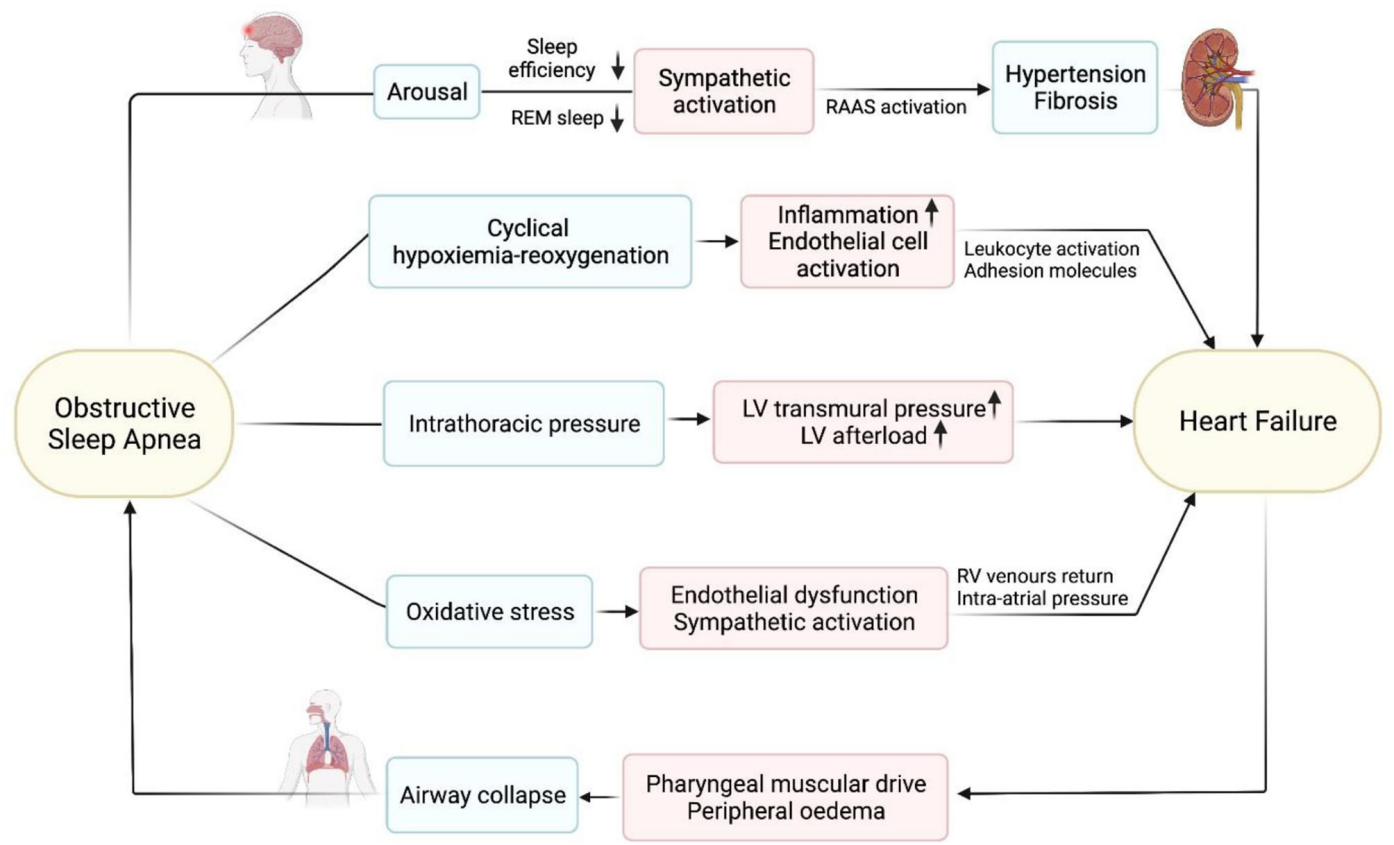
The pathogenesis of OSA in heart failure.

OSA is characterized by recurrent upper airway collapse and narrowing while sleeping (35, 36). Collapse is caused by motor neuron dysfunction, which directs the pharyngeal musculature (37, 38) (Figure 1). OSA could increase the possibility and severity of HF and affect the progression of HF through a variety of ways (e.g., significant oscillations in the ventilatory drive) which may result in periodic breathing to upper airway narrowing and collapse (39, 40). Intrathoracic pressure decreases when inhaled against a closed airway, which increases venous return to the right ventricle (41). This will increase in heart preload, which could promote a displacement of the interventricular septum toward the left side, further decreasing the LV function (42, 43). The negative intrathoracic pressure during OSA increases LV transmural pressure, which raises afterload (44). In HF patients, these repeated episodes of obstructive apnea cause an increase in sympathetic activity, as shown by elevated plasma catecholamine levels (45). Endothelial dysfunction, increased inflammatory markers concentration and blood pressure are caused by the recurrent occurrence of apneas and hypopneas (46–48). CSA appears to be secondary to HF and when carbon dioxide partial pressure (PaCO_2_) decreases below the apnea threshold (49). Due to pulmonary congestion stimulates the pulmonary vagus nerve and increases central and peripheral chemical sensitivity, patients with heart failure tend to hyperventilate for a long time (50–52). CSA occurs When the apnea threshold increases during the transition from wakefulness to sleep, or sudden increase in ventilation triggered by a spontaneous awakening PaCO_2_ to drop below the apnea threshold (53). The apnea continues until PaCO_2_ raises above the apnea threshold. Then ventilation will recover, ventilatory overshoot will occur, PaCO_2_ will go down below the apnea threshold, which is related to the increase in arousal and chemical sensitivity during the ventilation phase, which is characteristic of HF patients in CSA (52, 54).

Considering the high prevalence and significant clinical impact of SDB, it should be investigated in all HF patients. Although overlap could occur, if the hypoxias are mainly obstructive, it is recommended that doctors treat HF patients with SDB as OSA. If the hypoxias are mainly central, it is advised as CSA. Symptoms including worse sleep, nocturnal breathlessness, daytime sleepiness, nocturia, headaches in the morning, and decreased concentration and memory should be used as incentives for further investigation. Initial diagnosis usually requires PSG in a sleep lab. Recent studies have shown that home-based diagnostic and monitoring systems are also effective for high pre-test probability patients than traditional polysomnographic diagnoses. In addition, home-based methods for diagnosis and treatment could promote remote monitoring and facilitate greater compliance (55). The German Society for Sleep Research and Sleep Medicine is one of the first international associations to apply portable monitoring devices to patients with symptomatic CV diseases for CSA. This therapy could help improve the pre-test probability in patients with high risks for CSA, but it cannot clearly diagnose or rule out CSA, which requires PSG (56). Portable or home monitors cannot diagnose accurately. OSA can be diagnosed if PSG shows at least five obstructive-type hypoxemic episodes per hour (57). Patients can be defined as OSA or CSA if their events are more than 50% obstructive or central, respectively. Overnight PSG is the current gold standard diagnostic technique for SDB. This requires real-time inspection with non-invasive methods of sleep electroencephalogram, chin electromyogram, eye movement, heart rhythm, body position, oronasal flow, oxyhemoglobin saturation, and detection of respiratory effort that can distinguish between central and obstructive events. An alternate technique is to use intraoesophageal pressure as a measure of respiratory effort. It is, however, less commonly used. The influence of SDB on CV activity is measured by using pulse transit time, or the time it takes to move pulse waves to peripheral arteries. Pulse transit time may also be utilized to assess people with HF and SDB, and when coupled with capnography, it could give better results (58). A formal sleep study is needed to diagnose CSA in HF patients accurately. Differentiating central and obstructive hypopneas during polysomnographic scoring is strongly recommended to diagnose CSA properly.

Taken together, the treatment of comorbidities and optimization of risk factors in patients with HF have received more and more attention. This literature review aims to discuss current OSA treatments of published trials and briefly summarize CSA therapies to identify future treatment directions for SDB in patients with HF.

## Treatment

The core treatment in HF patients with SDB is optimizing medical management of underlying HF (59). Patients with moderate or severe OSA and accompanying comorbidities require OSA treatment. Treatment options for OSA include behavioral, medical, dental and surgical procedures (Table 1) (12). Pathological and therapeutic differences between OSA patients with HF and general population are shown in Table 2. All OSA patients should avoid alcohol and sedatives. So far, the efficacy of pharmacotherapeutic treatments for OSA is unclear (60). The 2013 ACC/AHA guidelines recognize the benefits of using CPAP to treat OSA in HF patients (61). CPAP has been reported to improve LVEF and quality of life and decrease sympathetic activity in patients with OSA and HF (62). Alternative treatments can be used for patients with mild-moderate OSA, but efficacy is less reliable than PAP therapy and lack beneficial effects on pre-and post-load.

**Table 1:** Current treatments for patients with OSA and HF.

**Treatment**	**Advantages**	**Limitation or disadvantages**
**Treat HF**		
Pharmacological therapy	Reduction in HF hospitalizations	No improvement in mortality
Device therapy	Reduce hospitalizations and mortality; improve cardiac remodeling and physical capacity	Swelling or bruising where the device is placed; bleeding; infection; heart rhythm problems.
Novel approaches	Significant reduction of the main endpoint of death	Need further investigation
**Nonsurgical treatments**		
**PAP therapy**		
CPAP	Reduction in AHI, sleepiness, blood pressure, depression, snoring and cardiovascular disease risk; very effective; improve respiratory function; good for weight loss	Poor tolerance; result in mucous membranes dryness, stuffy nose and skin allergies.
Bi-level PAP	Improving compliance; more sophisticated settings can be customized to user's specific needs; effective for users with moderate to severe obstructive sleep apnea.	More expensive than CPAP; easily lead to CSA in people originally diagnosed with OSA.
**Lifestyle modification**		
Weight loss	Good effects on metabolic and cardiovascular diseases.	Many patients cannot persist; it is effective for some patients.
Exercise	Good for weight loss; Positive effects for metabolic and cardiovascular diseases.	Not effective for all patients.
Positional therapy	Most patients can afford it.	Mainly applicable to patients with positional OSA.
Smoking and alcohol avoidance	Reductions in AHI but also specify the reductions in REM apnoeas and non-REM apnoeas.	–
Oropharyngeal exercises	Reduced OSA severity; reductions in AHI.	Whether patient persists is unclear; poor follow-up.
Oral appliances	Well-tolerated.	Not effect for people with severe OSA and obesity; Can cause temporomandibular joint discomfort.
**Other treatments**		
Supplemental oxygen	Can be used safely and effectively for infants who cannot undergo surgery.	Airway collapse.
Acupuncture	Reduce AHI and ESS and improve LSaO_2_.	Some bias and heterogeneity; there are inherent limitations with using the AHI calculated from one night of sleep to categorize disease severity.
**Surgical treatment**		
UPPP	Involve partial uvula and part of the soft palate; increase the pituitary gland.	Low efficiency than PAP; postoperative pain; may cause velopharyngeal insufficiency; weight gain.
Tracheostomy	Most patients with OSA can be cured; ensure adherence to treatment.	Unacceptable cosmetic result; effect on speech; need for long-term care.
Hypoglossal nerve stimulation	Positive effect and well-tolerated.	High price; maybe cause various discomforts of the tongue.
Maxillomandibular advancement	Very effective; adherence to therapy is ensured.	Long recovery time; maybe result in malocclusion, poor cosmetic results, and facial numbness or paresthesia.
Phasic or staged surgical protocol for OSA	Minimal morbidity and is well-accepted by patients; long-term cure.	–
Adenotonsillectomy	Reduction in the AHI; improvements in quality of life and behavior.	Not to resolution of the underlying sleep disorder in the majority of obese children.
TORS	AHI reduction; significant improvement in quality of life; clinical efficacy and cost effectiveness.	Minor secondary bleed; dysgeusia; persistent odynophagia to solids.

**Table 2:** Pathological and therapeutic differences between OSA patients with HF and general population.

	**Patients with OSA**	**Patients with OSA and HF**
Pathophysiological differences	Narrow pharynx related to fat accumulation; loss of pharyngeal dilator muscle tone causes complete or partial pharyngeal collapse at sleep onset.	Fluid accumulation in the legs while upright during the day could shift into the neck when recumbent during sleep; distension of the neck veins and/or edema of the peripharyngeal soft tissue; increase in peripharyngeal tissue pressure, predisposing to pharyngeal obstruction.
Therapeutic differences	Devices such as PAP, CPAP, BiPAP. Oral Appliances such as MAS; Surgeries such as Tracheostomy, UPPP, Maxillomandibular advancement and Adenotonsillectomy; life style modifications such as weight loss, positional therapy and behavioral therapy.	General OSA therapy; Pharmacological therapy and device therapy.

### Treat HF

Worsening of HF may increase the risk of resulting in more obstructive and central apneas. HF can exacerbate or reveal latent OSA by increasing upper airway instability, particularly when patients sleep in supine due to cervical venous congestion (63). One research discovered a correlation between volume shift from the lower extremities during sleep and AHI in HF patients with OSA (2). Volume overload might cause cervical venous congestion, exacerbating OSA (64). Therefore, the most necessary treatment is to optimize HF therapy. Optimizing HF therapy lowers preload and interstitial pressure in the lungs, which reduces the hyperventilation that causes OSA. Preload reduction could decrease cervical venous congestion and upper airway instability simultaneously (63). Since patients with HF are accompanied by fluid retention and rostral fluid shift, treatment strategies that decrease intravascular volume and venous congestion are more likely to reduce the severity of OSA and CSA.

Pharmacological therapy is the cornerstone of HF guidelines-based treatment. Beta-blockers and ACEIs can improve cardiac output and offer the symptomatic benefits of OSA (65, 66). Diuretics can effectively reduce OSA severity by preventing fluid retention and reducing mouth fluid transfer (67). Three-day administration of spironolactone and furosemide result in an increase in upper airway caliber and a decrease in AHI (p < 0.001) in patients with diastolic HF and severe OSA have been reported by Bucca et al. (68). Complications of HF deserve particular attention. HF pharmacological can help with OSA by lowering volume shifts and volume overload into the lungs and cervical regions (69). Cardiac resynchronization therapy can improve CSA in patients with CHF by reducing AHI, but no significant reduction has been found in subjects with OSA (70). Since these early studies, the treatment of heart failure has undergone significant changes, and the prognosis of heart failure has improved (71). This may have an impact on the prevalence and characteristics of SDB in CHF patients. Guidelines therapy for HF is a standard of care in current HF practices worldwide, and the treating HF team refines it regularly. As a result, the role of HF optimization in the treatment of newly diagnosed OSA in patients with persistent HF is unclear. On the other hand, HF optimisation may be relevant for selecting whether or not to initiate positive airway pressure (PAP) device therapy in patients with SDB. They are not currently on optimal guideline-based HF therapy. Once optimum HF treatment has been determined, it may be desirable to assess these individuals' severity and type of SDB.

### Non-surgical Treatments

#### PAP Therapy

The crucial OSA treatment is CPAP, which splints open the upper airway to improve patency during sleep. Using a CPAP machine regularly reduces sleepiness symptoms in patients with moderate-to-severe OSA and promotes quality of life metrics (72). With a success rate of around 75%, CPAP has ranged the preferred treatment option for moderate to severe OSA. Unfortunately, recorded rates of non-adherence, defined as utilizing CPAP for 4 h or more every night, ranging from 46 to 83%. However, patients who oppose or are unable to tolerate CPAP require other treatment options (73). The 2017 guidelines ACC/AHA/HFSA recommend using CPAP in patients with HF and OSA to improve sleep disorder symptoms (74). CPAP treatment can improve SDB and the left ventricular ejection fraction and change sympathetic nerve activity in patients with OSA (75). CPAP treatment can improve SDB and the left ventricular ejection fraction and change sympathetic nerve activity in patients with OSA. When considering compliance with CPAP, the adhering group had a better prognosis (76).

According to small pilot studies and observational studies, CPAP appears to improve cardiac function, blood pressure management, and reduce CV events in people with HF (77–79). However, the impact of using CPAP to treat OSA on CV events was studied in a recent large RCT. The study of Sleep Apnea Cardiovascular Endpoints (SAVE) was a multicenter, multinational, randomized trial examining whether CPAP may assist people with OSA to avoid major CV events (80). The trial assigned randomly 2,717 individuals between the ages of 45 and 75 with moderate-to-severe OSA and coronary or cerebrovascular illness to receive either CPAP + usual therapy or usual care alone. There was no significant benefit in avoiding CV events, myocardial infarction, stroke, hospitalizations for unstable angina, HF, or transient ischemic attack after a mean follow-up of 3.7 years. The average AHI in the CPAP arm, on the other hand, reduced from 29 to 3.9 events/h, indicating that it improved quality of life indicators such as reducing tiredness and decreased depressive symptoms. The average CPAP use was more than 3 h/night, which might not be enough to enhance the CV outcomes. Those patients who were not very sleepy were included in the trial, and thus might have a lower chance of benefiting from the CPAP impact on OSA-related CV outcomes (81). Other flaws in the SAVE study could have influenced the outcomes. The trial's participating sites had substantial variations in practice, and the study was underpowered to provide enough evidence on the impact of CPAP on secondary CV endpoints. There are many studies on CPAP for OSA, some of which have specifically studied HF. In an RCT involving 55 patients with OSA and HF, 3 month of nocturnal CPAP improved LVEF and reduced urinary noradrenaline excretion (82). Colish showed compared with those treated with CPAP, and there was a significantly higher rate of hospitalization or death in the non-CPAP group of patients with HF and moderate to severe OSA (83). This result is similar to the other two studies (84, 85). Debates on the effect of CPAP on the risks of stroke and myocardial infarction are not in this review's scope.

Bilevel positive airway pressure (bilevel PAP) could adjust to different levels of inhalation and exhalation. Bilevel PAP is suitable for treating OSA patients with pressure more than 15 cm H_2_O with obesity, hypoventilation syndrome and central hypoventilation (86). However, no studies have shown that the adherence or effectiveness of bilevel PAP is better than CPAP (87). One preliminary pilot study showed bilevel PAP ventilation could improve HF and concomitant OSA (88). Nevertheless, a trial showed that bilevel PAP could improve CV and respiratory functions and provide more benefits (89). These require larger and more trials to confirm these preliminary results. Khayat et al. identified that BiPAP could improve heart function and distance in the walking test (88). According to Mehta et al., BiPAP has a rapid effect on improving gas exchange, tidal volume, and adherence for HF patients (90).

#### Lifestyle Modification

In the United States, more than 65% of adults are overweight (BMI > 25 kg/m^2^) (91). Excess body weight is the main risk factor for OSA (92, 93). Research supports the relationship between weight loss and OSA severity, and it is likely to have the same effect as OSA in HF patients (94, 95). In one randomized controlled trial (RCT), the effect of weight reduction accomplished by gastric banding vs. proper medical treatment on OSA was investigated (96). Patients who received laparoscopic gastric banding exhibited a similar reduction in AHI as the control group after 2 years of follow-up. Still, only the treatment group improved secondary outcomes like sleepiness, quality of life, and the 6-min walk test. Losing weight can help OSA patients obtain more from their CPAP. Weight reduction led to incremental improvements in serum triglyceride levels, insulin resistance, and blood pressure in a randomized study of 90 people with OSA who utilized CPAP (97). It should be mentioned that CPAP treatment for OSA has been linked to slight weight gain (98). In multiple studies, weight losses caused by a series of dietary and lifestyle changes are associated with a significant decrease in AHI (99–101). A 3% increase/decrease in AHI was associated with a 1% gain/loss in body weight, and a 10% weight increase was associated with a 6-fold increase in the risk of AHI more than 15 events/h in the Wisconsin Sleep Cohort (94). Systematic reviews and meta-analyses showed weight loss support this relationship both in surgical and non-surgical patients (101). Although OSA severity has generally improved, it may not be possible to eliminate OSA after weight loss. Then, the fundamental questions about why weight decrease could improve OSA and why weight increase exacerbates OSA are still unanswered. Figuring out these questions is crucial for optimizing clinical management and personalized treatments of OSA.

Regardless of weight fluctuations, exercise training and physical activity are associated with decreased OSA and improved sleep efficiency (102). In a meta-analysis of five investigations involving 129 people, the pooled estimate of AHI decrease with exercise was 6.3 events/h (~32% reduction) (103). The mechanisms by which exercise improves OSA remain unclear; however, they potentially include reducing rostral fluid redistribution, stabilizing chemoreceptor sensitivity, enhancing the strength of pharyngeal dilator muscles, nasal resistance, improving sleep quality, and losing weight. A decrease in a rostral fluid shift during sleep with a subsequent rise in pharyngeal volume resulted in AHI change. Physical exercise may have this effect via stimulating the musculovenous pump that prevents the accumulation of the pharyngeal fluid in the supine position by counteracting fluid accumulation in the legs. Forty-five minutes of exercise twice a week lowered AHI from 58 to 40 per hour of sleep in one study without inducing weight loss (104).

Positional therapy (i.e., the avoidance of lying on the back during sleep) is a not common treatment. Still, itseems to be an appropriate form of treatment with postural OSA patients (105). Most patients with OSA breathe abnormally when sleeping in the supine posture. Sleeping in the lateral position may have a marked reduction in AHI (106). Positional modification treatment includes using backpacks, binders, and tennis balls linked to vests or braces, as well as electronic sensors with alarms or vibrations to notify a client to change positions. In the largest research to date, compared with the lateral position, approximately 55% of patients had twice as much abnormal breathing in the supine posture (106). Patients with mild to moderate OSA (ranging from 65 to 69%) have a higher prevalence of posture patients than severe OSA (107). Compared to CPAP, a systematic review and analysis of positional treatment indicated that it had a limited effect on this intervention (108). Positional therapy's efficacy is likely to be significantly reliant on the intervention used. Irrespective of the impact size, this intervention can help patients who cannot tolerate CPAP.

The upper airway dilator muscles are essential for keeping an open airway during sleep (109); numerous recent studies have looked into the impact of oropharyngeal workouts on the severity of OSA. Guimaraes and colleagues allocated randomly 31 patients with moderate OSA to either a sham treatment (*n* = 15) or a series of oropharyngeal exercises adapted from speech therapy (*n* = 16). The exercises comprised isotonic and isometric tongue, soft palate, and lateral pharyngeal wall motions. According to the AHI and the lowest oxygen saturation measured by polysomnography, 3 months of exercise training lowered OSA severity by 39% (110). One recent meta-analysis and systematic review on OSA found nine adult studies (120 patients) that evaluated the effect of oropharyngeal exercises (also quoted as myofunctional therapy) (111). The adult patients had moderate OSA, majorly, and group data revealed a 50% reduction in AHI on average. Because the oropharyngeal exercises follow an integrative strategy, it is impossible to determine the impact of each patient exercise on the total result. More significantly, the patient must practice the workouts regularly (2 or 3 times per day), limiting their clinical applicability. As a result, In “real-world” clinical practice, it is unknown how successfully patients respond to oropharyngeal exercises (112).

Alcohol is a central nervous system depressant with peripheral muscle relaxation. It can play an essential role in regulating the incidence and severity of OSA. The current American Academy of Sleep Medicine (AASM) guidelines recommend that patients care about the potentially harmful effects of alcohol on OSA (113). Drinking alcohol can significantly worsen AHI and mean SpO_2_. This was especially significant in patients with a history of OSA and snoring. Alcohol also seems to lead to prolonged respiratory and lower minimum SpO_2_ (114). Patients diagnosed with OSA and those at risk, especially snorers, should understand the possible effect of alcohol on breathing in sleep. Alcohol consumption should be considered as a changing risk factor for OSA (115). Other CV diseases and diseases related to peripheral arteries can also be affected by drinking. Mild-to-moderate alcohol consumption is associated with a lower incidence of ischemic stroke. On the other hand, alcoholism is associated with an increased risk of bleeding and ischemic stroke. Arrhythmias is related to alcoholism, even in men without CV disease (116). Individuals with untreated OSA should avoid alcohol since it prolongs and increases the number of obstructive respiratory episodes during sleep and the degree of oxygen desaturation that occurs during these events (117).

In conclusion, weight loss, exercise training, oropharyngeal exercises, positional therapy and alcohol avoidance could be beneficial adjuncts or alternative treatments for people who suffer from OSA. The magnitude of these therapies' effects and their capacity to reduce the CV implications of OSA remain unknown. It is vital to remember that these treatments should not be utilized as a stand-alone solution for OSA. These therapies on an individual basis can be considered in CPAP-intolerant patients with OSA, and patients should be regularly followed.

#### Oral Appliances

Oral appliances (OAs) can improve the OSA index, which was measured by polysomnography. Compared with the baseline values in a sleep trial, oxygen saturation, arousal index and sleep efficiency were significantly improved (118). More than 65% of OA-treated patients have reduced AHI by more than 50%, and at least 33% achieve a complete remission (119). OA compliance is claimed to range between 40 and 80%, and the majority of the patients prefer OA treatment to surgery or CPAP. CPAP provides higher PSG outcomes than OAs, especially when lowering AHI. As a result, CPAP has a higher success rate in treating sleep apnea. Nevertheless, in terms of clinical and associated implications, CPAP and OAs are similar. According to the American Academy of Sleep Medicine (AASM) practice recommendations, OA is the favored treatment for OSA when CPAP is ineffective, regardless of severity (120). A wide variety of OA devices and designs have been employed to treat OSA. Tongue-retaining devices and mandibular progressive and orthodontic appliances are the two most frequent designs. An OA improves the upper airway by changing the position of the tongue and other upper airway components. The American Academy of Dental Sleep Medicine and the AASM claim that the recommended OA is a tailored, titratable, tooth-borne appliance designed to advance the mandible. Individuals with particular craniofacial characteristics, such as a short minimum retroglossal airway, a low height of an anterior face, mandibular retrusion, respond to OA treatments effectively (121). In recent years, devices are emerging which rely on computer-aided design or computer-aided manufacturing technology. Long-term use of OA demands meticulous dental and bone monitoring. Despite this, OAs do not cause major skeletal changes of mandibular rotation, according to a recent comprehensive review and meta-analysis. The use of OA for a long time has been associated with changes in the mouth. A considerable alteration causes reduced overbite and overjet in mandibular incisor angle. Despite the severity of these adverse effects, the benefits of utilizing an OA outweigh them, especially given the potentially fatal nature of OSA. Patients must be warned of their treatment's potential long-term negative consequences before starting it (122). Digital intraoral scans can design appliances and replace the traditional impressions, and digital manufacturing techniques are increasingly being used. These can streamline the process and result in more rapid access to treatment and better appliances, improving acceptance and efficacy (123).

#### Other Treatments

Low-flow supplemental oxygen can be used safely and efficiently on newborns who cannot undergo surgery and for whom CPAP is not an option due to a lack of adequate interface. Because it is not curative, it is only a temporary treatment. There was a drop in AHI from 18 to 3 on oxygen and an increase in oxygen saturation in infants with central, obstructive, and mixed apneas with a mean age of 4.8 months. Low-flow supplemental oxygen through nasal cannula was classified as 0.25 to 1 L/min. Supplemental oxygen, in addition to perhaps supplying a small amount of positive airway pressure, is thought to reduce the diaphragm's contracting force with the correction of hypoxemia and the related increased respiratory drive, which can contribute to airway collapse. Furthermore, treatment of hypoxemia may improve geniohyoid muscle tone (124). Supplemental oxygen therapy can be used for abolishing intermittent hypoxia in OSA. If intermittent hypoxia is the main reason for increased daytime blood pressure rather than repeated arousals, it can decrease the blood pressure during the day (125). Two previous RCTs showed no supplemental oxygen effect on blood pressure in OSA (126, 127). But their study both had limitations; the oxygen flow rates were too low at 2.5–3 L/min, and the most severe hypoxemia and OSA patients were excluded, and the average amount of supplemental oxygen used was modest. These trials have not yet clearly determined whether repeated arousals or intermittent hypoxia are the main cause of elevated blood pressure during the day in OSA due to these limitations.

Acupuncture helps patients with OSA reduce their AHI and the amount of nocturnal respiratory episodes (128), which may have a quicker therapeutic effect on OSA Patients Acupuncture may also help OSA patients reduce their AHI and Epworth Sleepiness Score (ESS) and increase their lowest oxygen saturation (LSaO_2_). There were several drawbacks. To begin with, there is some bias and heterogeneity in acupuncture treatment for OSA. Acupuncture's varied interventions could be one of the explanations. Second, patients with OSA were classified as mild, moderate, or severe before treatments based on their AHI scores. The AHI is a useful tool for diagnosing and categorizing the severity of OSA patients' conditions. However, utilizing the AHI determined from one night of sleep to identify disease severity has inherent limitations. The AHI is impacted by various factors and can vary over time and even between successive nights. Third, the included RCTs' methodological quality was generally low. The majority of the studies examined, for example, had a high potential for performance bias (60).

### Surgical Treatments for OSA in HF

#### Uvulopalatopharyngoplasty (UPPP)

UPPP, which comprises the excision of the uvula, tonsils, and posterior velum, is one of the most common surgical therapies for OSA. It consists of a tonsillectomy and excision of the uvula and part of the soft palate. UPPP corrects retropalatal obstruction but does not address retroglossal obstruction. The success rates for UPPP are only ~40–50% (129, 130). The AASM does not suggest UPPP as a stand-alone treatment for moderate to severe OSA because it does not consistently result in AHI normalization. Friedman stage I (big tonsils and rather normal palatal position) in a meta-analysis was found to be the only surgical success indicator. In contrast, Friedman stage III and low hyoid position were discovered to be surgical failure indicators (131). Despite only partial reductions in the AHI and limited success as a stand-alone cure for OSA, UPPP remains an important therapeutic option for many patients refusing or intolerant of CPAP. Prior studies have primarily focused on determining efficacy and best patient selection for UPPP, and have not evaluated the effects of the surgery on reducing the incidence of CV complications (132). OSA patients with AF, diabetes, or dyslipidemia had varying results after UPPP. For example, UPPP significantly reduced the incidence of HF in patients without diabetes compared to patients with diabetes. Also, AF was less likely to occur after UPPP than in diabetic patients. It is possible that diabetes, which is often associated with obesity and limited physical activity, contributes to OSA, further increasing the risk of CV disease (133). These differences in outcomes after UPPP are probably due to the different etiologies of CV disease.

#### Tracheostomy

From the late 1960s to the early 1980s, Tracheostomies were the most common surgical method for OSA patients' treatment when traditional medicinal therapy failed. Even though tracheostomy offers the advantage of upper airway blockages bypassing and can greatly treat OSA, it is regarded as a last-resort surgical procedure. Tracheostomy is considered the most effective therapy for refractory OSA patients with severe hypoxemia. The literature researching results of tracheostomy for adult patients supports this claim. Almost all patients report improvements in daytime sleepiness and fatigue (134). Because of tiny numbers in this category, there was a lack of research examining patients who underwent tracheostomy for this indication in children. Patients who have not responded to the medicinal treatment, are not soft tissue surgery candidates, and/or have declined maxillomandibular advancement (MMA) surgery are appropriate candidates for tracheostomy (135). In adults, it could be regarded as short-term therapy to protect the upper airway after invasive upper airway surgery or as a permanent treatment to alleviate OSA. OSA and morbid obesity are usually a last resort and are a complex process. However, it did reduce the morbidity and mortality of these patients. Compared with the burden of living with severe OSA, the quality of life is greatly improved during this process and is well-tolerated (4).

#### Hypoglossal Nerve Stimulation

According to researchers, hypoglossal nerve stimulation has been developed and applied in OSA treatment. Pilot experiments in humans and animals demonstrated the feasibility of this method, paving the way for the creation of fully implanted pacemakers for therapeutic use. The method was then shown to be safe and effective in feasibility experiments using a variety of simulation platforms. The first feasibility study using distal nerve stimulation in 8 patients was undertaken with moderate to severe OSA, and substantial decreases in apnea-hypopnea indices were seen (136). Following that, two single-arm interventional trials using similar technologies were done on a larger number of patients, and both replicated comparable reductions in AHI (137). One of these devices has already been approved by the FDA, while another is now through pivotal testing. Clinical trials have generated valuable information that can be used to improve therapy responses. These include criteria for selecting patients and procedures for stimulating various parts of the hypoglossal nerve and/or genioglossus muscle. New neurohumoral and molecular techniques for promoting efferent and afferent motor circuits are being tested (138).

#### Maxillomandibular Advancement Surgery

According to comprehensive research, MMA is an effective treatment for obstructive sleep apnea because it enlarges the upper airway in the anteroposterior and lateral planes and raises the hyoid bone (139). The traditional MMA surgical method combines regular Le Fort I osteotomy along with a mandibular sagittal split osteotomy to advance the maxilla and mandible. The soft palate and the tongue base are pushed forward, allowing more air to pass through and lowering upper airway resistance (140). Because Asians have a bimaxillary protrusion, flat nose, and a weak chin before surgery, the aesthetic effects of MMA treatment for Asian patients with OSA should be assessed (141). In previous research, researchers found that segmental maxillomandibular rotational advancement (extrusion of the anterior segment, elongation of the posterior maxilla, and counterclockwise rotation of the maxillomandibular complex) is a successful treatment modality for OSA in an Asian population with a convex craniofacial profile and endoskeletal Class II malocclusion. Because it has no side effects on facial appearance or dental alignment, modified MMA is a successful treatment for moderate-to-severe OSA (142). Surgical-orthodontic integration is necessary for better results. Early improvement can be achieved with the surgery first strategy.

#### Phasic or Staged Surgical Protocol for OSA

Surgical therapy is a potentially curative treatment choice. To enable the use of a surgical protocol that improves clinical outcomes, a thorough presurgical evaluation with the identification of the kind of airway anomaly is required. The goal of Phase I surgical regimen is to use particular surgical methods to remove the obstruction (s). Airway reconstruction should be approached methodically and step-by-step to reduce surgical interventions and avoid unnecessary surgeries. A risk-management approach will help to reduce treatment problems while still attaining cure. It is now well-known that the lateral pharyngeal wall, soft palate, tongue base, or hypopharynx are all potential sites for upper airway obstruction in OSA patients. The blockage level governed in the presurgical assessment determines the surgical therapy for Phase I. Surgical options for oropharyngeal obstruction include UPPP and/or genioglossus advancement with hyoid myotomy or suspension for base-of-tongue blockage (143). A repeat PSG is performed around 6 months after surgery, and individuals who do not achieve surgical effectiveness or cure are referred to phase II surgery. For phase I failures, phase II surgical reconstruction is reserved and comprise of MMA advancement osteotomy (144).

#### Adenotonsillectomy (AT)

In general, studies examining the effectiveness of AT as an OSA treatment show that AHI lowers in both obese and non-obese children who receive AT. A lower AHI is generally regarded to be beneficial to the newborn. Obese children, on the other hand, appear to benefit from AT less. Obese children are compared to children of average weight in a study to show that OSA in obese children is far more susceptible to recur after surgery (145–147). Apostolidou et al. were the only researchers to find no difference between obese and non-obese youngsters in post-operative AHI (148). The explanation for this is uncertain because both sets of children were of an adenotonsillar size and identical age. There was a significant difference in the performance of AT between obese and non-obese children with OSA in related research with children of the same age range (148). According to Mitchell et al., before surgery, Obese children were more likely to have severe OSA than normal-weight children; however, after adjusting for the pre-operative AHI, obese children had a higher chance of OSA persisting of about 4.7-fold after AT than children of normal-weight (146). According to O'Brien et al., obesity had an adjusted 3.7 odds-ratio for persisting OSA after AT (145), implying that, regardless of the severity of the original diagnosis, Obesity is a risk factor for OSA development independently. It's worth mentioning that the majority of the children in these studies had enlarged tonsils and were referred for AT. The most common way to estimate the size of a child's tonsil is to use a four-point scale. However, it is well-established that current grading scales do not accurately reflect the underlying severity of OSA (149).

#### Trans Oral Robotic Surgery (TORS)

TORS was first published in 2010 as a treatment for OSA that included open tongue base reduction and hyoid epiglottopexy (150). The method allows for multi-planar tissue transactions from any angle. This approach has been shown in several studies to increase surgical access to the base of the tongue (BOT). TORS is the newest tool in the arsenal of otorhinolaryngologists—Head and Neck Surgeons in the fight against OSA (151). TORS is suited for multilayer treatment of OSA because of its superior visualization and ergonomics. However, not all patients are candidates for TORS, and its efficacy in obese patients is debatable. Given the global obesity epidemic, this is a critical subject that must be addressed as soon as possible. Despite lower success rates as BMI rises, TORS has a success rate of more than 50% in non-morbidly obese patients (BMI 30–35 kg/m^2^) (152). A 50% success rate may appear poor at first glance, but keep in mind that this is a patient cohort with a life-threatening condition and no other option but a tracheostomy. As a result, TORS is a vital therapy option for non-morbidly obese OSA patients who have failed to respond to other treatments (150).

### Treatment of CSA in HF

There are several options in patients with CSA and HF treatment. The treatment of HF itself could improve the prevalence of CSA. CRT can improve AHI in patients with CSA in patients with HF (70). The frequency of CSA can be lowered by LVAD implantation and cardiac transplantation (153). Garrugue et al. first proposed CRT to treat SDB in 2002 (154). After that, a number of studies have associated CRT with a significant reduction in AHI in patients with HF and CSA (155, 156). A meta-analysis found that CRT had a significant reduction in AHI in 133 CSA patients, but no significant change in AHI was found in 81 OSA patients (70). Acetazolamide has been found as a possible treatment for metabolically induced CSA. In small trials, it is related to lowering patients' AHI and improving O_2_ saturation and sleep quality (157, 158). Nocturnal supplemental oxygen can increase the physiologic apneic threshold and is considered a treatment to decrease CSA (159). Supplemental oxygen has been reported to reduce periodic breathing and sympathetic compensation characteristic of CSA with significant improvement of nighttime oxygen and normalizing PaCO_2_ (18).

There are two common ways of non-invasive positive pressure ventilation (PPV) to improve CSA: CPAP provides a stable positive pressure, and adaptive servo ventilation (ASV) can effectively adjust pressure support and backup rate to make the breathing pattern relatively normal (160). Because patients with HF and CSA are related to increased LV filling pressures, CPAP could be used for improving hemodynamics. However, the effects of CPAP on CSA have not been consistent. This is probably due to differences in how it is applied (161). Clinical trials and meta-analyses showed, ASV can effectively suppress CSA events and has been proven to improve patients' heart function and quality of life (160, 162–165). Although PAP effectively treats CSA/CSR, there is no conclusive evidence to reduce mortality. The SERVE-HF trial (166) (Treatment of Sleep Disordered Breathing with Predominant Central Sleep Apnea by Adaptive Servo Ventilation in Patients with Heart Failure trial) investigated whether PPV has adverse effects on hemodynamics in patients with severe systolic HF. Due to the unexplained increased risk of death in the ASV group of patients with systolic HF and CSA in the SERVE-HF trial, this recommendation proposes not to start ASV in patients with an LVEF <45%. Transvenous unilateral phrenic nerve stimulation (PNS) has been reported to be an effective and safe treatment in patients with CSA (167). TPNS mainly involves a nerve stimulator with traction and sensing leads, usually landing in the right thoracic area. The leads enter the right brachiocephalic vein to stimulate the phrenic nerve, and the sensing lead is usually placed in the azygos vein to sense breathing by thoracic impedance. The system is normally activated after 1-month implantation, and the device is gradually and automatically programmed for more than 3 months to capture the whole diaphragm and contraction during sleep (157, 168).

## Conclusions

OSA has adverse effects on heart function and could be related to the occurrence and progression of HF. It is independently associated with an increased risk of mortality and morbidity. The diagnosis and treatment of OSA could be difficult. Symptoms of OSA cannot be easily noticed, and up to 80% of patients are still undiagnosed. Even the gold standards of diagnosis and management have significant disadvantages. PSG is inconvenient and expensive, and CPAP therapy is often affected by intolerance and general non-compliance. The published studies on the prognosis of CV following surgical treatment of OSA is limited, and the quality is generally poor. Data provide from small research indicate that surgical therapy may improve heart function. Although our understanding of OSA and HF has improved, there are still unresolved problems and suggested treatment options. Larger follow-up and constant RCTs with more rigorous research are needed.

## Author Contributions

YW and TP were responsible for the manuscript concept and design. YW, CS, and TP prepared the manuscript draft and contributed to critical revision of the manuscript. YW prepared the figure and table. All authors contributed to the article and approved the submitted version.

## Funding

YW was financially supported by the China Scholarship Council (CSC) for her MD study in Sleep Medicine Center, Charité Universitätsmedizin. The CS had no role in the design or conduct of this research. YW is a mentee of World Sleep Society's International Sleep Research Training Program (ISRTP) 2021. TP was partially supported by a Russian Federation Government Grant No.: 075-15-2019-1885.

## Conflict of Interest

CS was employed by GmbH. The remaining authors declare that the research was conducted in the absence of any commercial or financial relationships that could be construed as a potential conflict of interest.

## Publisher's Note

All claims expressed in this article are solely those of the authors and do not necessarily represent those of their affiliated organizations, or those of the publisher, the editors and the reviewers. Any product that may be evaluated in this article, or claim that may be made by its manufacturer, is not guaranteed or endorsed by the publisher.
